# Cemento-Osseous Dysplasia in a Female Bronze Age Skeleton (North Caucasus)

**DOI:** 10.1007/s12105-025-01767-1

**Published:** 2025-02-25

**Authors:** Julia Gresky, Melina Frotscher, Sophia Thiem, Alexander Stoessel, Alexey Kalmykov, Natalia Berezina

**Affiliations:** 1 Faculty of Biology and Psychology, Georg-August University, 37073 Göttingen, Germany; 2 Department of Natural Sciences, German Archaeological Institute, 14195 Berlin, Germany; 3 Institute of Zoology and Evolutionary Research, Friedrich Schiller University Jena, 07743 Jena, Germany; 4 Department of Archaeogenetics, Max Planck Institute for Evolutionary Anthropology, 04103 Leipzig, Germany; 5 Independent Researcher, Stavropol, 355006 Russia; 6 Research Institute and Museum of Anthropology, Lomonosov Moscow State University, Moscow, 125009 Russia

**Keywords:** Oral pathology, Fibro-osseous process, Growth/development, Paleopathology

## Abstract

**Purpose:**

The earliest known case of cemento-osseous dysplasia could be detected in a Bronze Age skeleton, dating back 4500 years ago in the region of the North Caucasus. Although the soft tissue was missing, sufficient diagnosis could be achieved by using different methods that prove the existence of fibro-osseous processes already in prehistory. Skeletal remains provide a direct view of such changes which cannot be obtained from a living patient without compromising.

**Methods:**

A skeleton of a 30-40-year-old female individual from the burial mound of Budyonnovsk 10 (including 19 individuals) in Southern Russia was investigated using macroscopic, radiographic, and microscopic methods.

**Results:**

In the mandible, destruction of the labial wall of the alveoli 32 and 31 is already visible macroscopically. At the base of the lesion, the original bone is replaced by fine porous bone including small dense particles: plain radiography and computed tomography evidence localized processes to the periapical areas of all lower incisors. The lesions are mainly radiolucent, only the particles in alveolus 32 have a radiopaque appearance. Microscopy shows woven bone as filling of the lesions and additional hypocellular materials in alveolus 32, which can best be explained as cementum-like structures.

**Conclusions:**

The lesion´s location in the periapical areas of the lower incisors, the woven bone, and cementum-like structures fit the diagnosis of periapical cemento-osseous dysplasia. The presence of a second individual with focal cemento-osseous dysplasia in this burial mound is an interesting co-occurrence that requires further genetic analysis.

**Limitations:**

The diagnosis is solely based on the skeletal remains, soft tissue components are missing.

**Suggestions for Further Research:**

Genetic analyses are planned to detect the underlying mutation for the two individuals.

## Introduction

The facial skeleton is prone to develop fibro-osseous lesions of various origins [1]. They differ according to their location in the skull, ranging from rare diseases like fibrous dysplasia and yet not well-defined small fibro-osseous processes in the entire area of the facial skeleton [2–5], to specific lesions of the tooth-bearing areas. These include odontogenic fibroma, cementoblastoma, different types of cemento-osseous dysplasia, and ossifying fibroma [1]. The special feature of odontogenic dysplasias, originating from the periodontal ligament, is the presence of cementum-like materials.

Although this substance is brittle, it can persist in human skeletons for thousands of years, still enabling a diagnosis in skeletons from archaeological contexts [6]. Here, we present a skeleton of a 30-40-year-old female individual who had lived in the northern Caucasus approximately 4500 years ago. The Stavropol region in Southern Russia comprises hundreds of cemeteries consisting of burial mounds dating from the Bronze Age to Medieval times. The soil and climatic conditions in this region favor skeletal preservation, therefore it was possible to detect distinct osseous changes in the mandible of this Bronze Age female. Another male individual from the same burial mound showed similar changes in the mandible. He was diagnosed with unifocal cemento-osseous dysplasia of the alveolus 43 [6].

Paleopathology is the study of diseases in past populations [7, 8]. Investigating skeletons from archaeological excavations, mummies, or bog bodies, this research aims to shed light on the general health status of single individuals or larger groups [9]. Furthermore, it focuses on the origins, distribution, and evolution of infectious diseases [10, 11] or the long-term perspective of specific disease groups, e.g. rare diseases [12, 13].

Investigation of fibro-osseous lesions in skeletons from prehistory could add to clinical results. The lesions are directly visible in defleshed bones, they can be documented by photography and digital microscopy without any restrictions providing a different view of these lesions. Furthermore, the often subtle changes can be detected more precisely in micro-computed tomography scanning without the risks of high voltage radiation to living patients. Large samples of the lesions can be taken and investigated in detail by microscopy or genetic analyses without creating any damage to the living.

By studying fibro-osseous lesions, we will be able to enlarge the sample size of certain lesions and detect possible changes in diseases during human history. Furthermore, by studying larger populations, we will be able to compare their distribution pattern in ancient and modern groups, their predilection for a certain age and sex, and with the help of genetic analyses provide information about the origin of the affected people.

The study aims to diagnose the pathological changes of the female’s mandible based on clinical literature and to compare them to other fibro-osseous lesions of the odontogenic apparatus. Furthermore, this finding will be contextualized with the second individual in the same burial mound having a fibro-osseous lesion.

## Materials and Methods

**Fig. 1:**
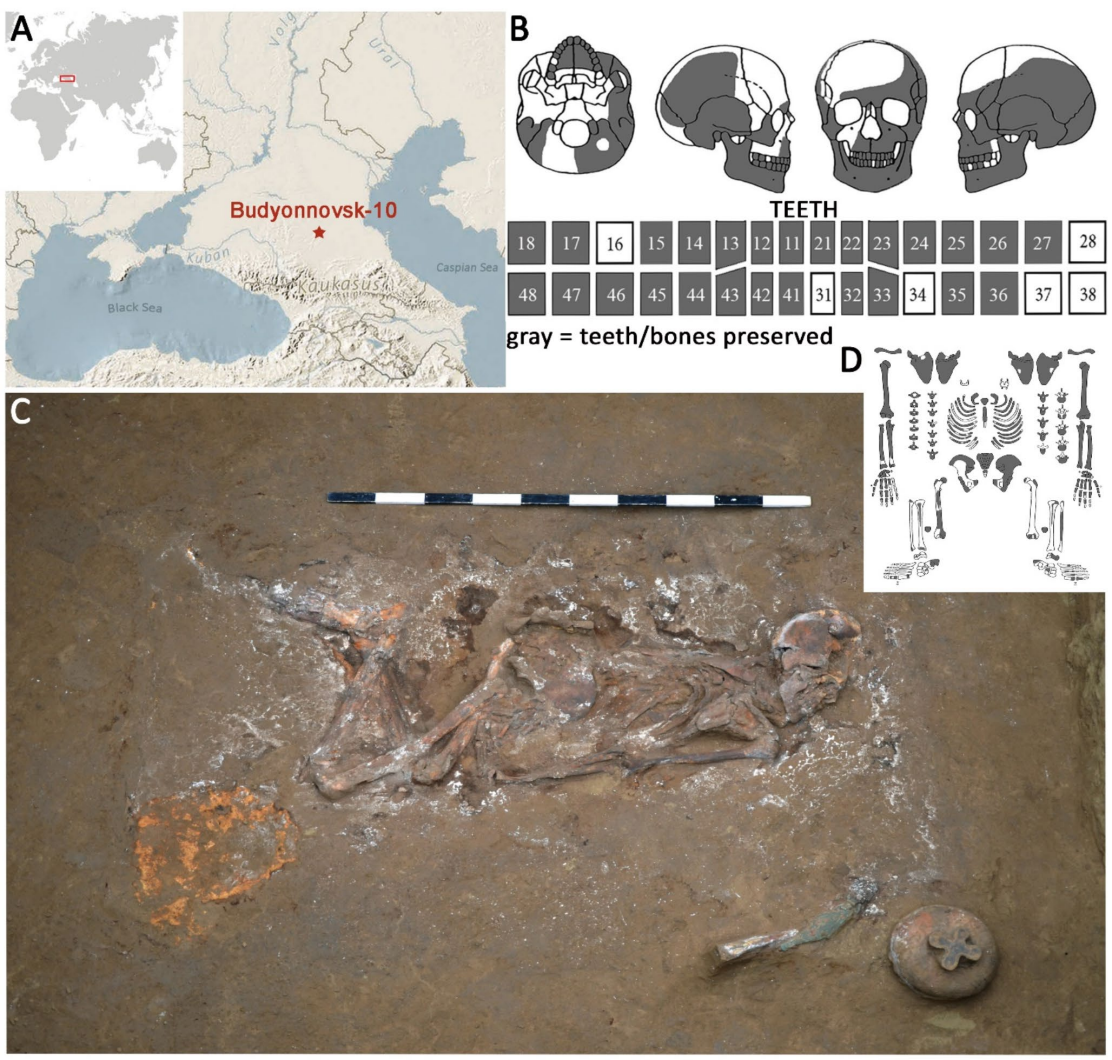
**A**) Map of Budyonnovsk, Stavropol region, South Russia. **B**) Skeletal inventory of the cranium and teeth, bones present for examination are marked in dark gray. Numbering of teeth after FDI scheme. **C**) The individual is placed in a crouched position on its left side in a Catacomb burial of Budyonnovsk 10, Burial mound 7, Burial 2. D) Skeletal inventory of the postcranium, bones present for examination are marked in dark gray. (Credits A, B, D: J. Gresky, C: A. Kalmykov)

In 2013 the large burial mound field with more than a hundred burial mounds at the banks of the river Mokraya Buivola in the steppe zone of the Stavropol region of South Russia was excavated (Fig. 1A). The vast majority of the mounds originated in the middle Bronze Age, but some burial mounds were used as cemeteries until the late Middle Ages.

This paper focuses on the site of Budyonnovsk 10, particularly on the burial mound 7, comprising 19 burials, of which the presented one has number 2. The individual of interest was buried in a single inlet burial in a catacomb. The burial was covered with its mound, indicating the high social status of the individual. The body of a four-wheeled wooden cart at the bottom of the pit contained the skeleton and most of its grave goods: two ceramic incense burners, a flint tool, a bronze needle in a bone case, a bronze knife, and a pile of beads. The individual was placed on its left side in a crouched position facing the SSW (Fig. 1C). Archaeological methods date the burial to the Middle Bronze Age, late Catacomb culture (2500 − 2300 BC).

The individual is almost completely represented, only the bones of the lower extremities are very fragmentary (Fig. 1B, D). The surface of the bones is well-preserved, and their consistency is rather fragile. Features of the pelvic morphology point to female sex while characteristics of the skull show a male tendency [14, 15]. This discrepancy is not uncommon as, in many individuals, not all present skeletal features indicate one or the other biological sex straightforwardly. Instead, these features have to be weighted for a reliable sex determination. In the presented case, pelvic morphology due to its physiological purpose, is more reliable than the features of the skull, in synopsis pointing to a female individual. For age estimation, dental wear [16], the morphology of the pubic symphysis [17], and the degree of degenerative processes of the joints were considered, suggesting an age between 30 and 40 years.

Investigation of the human remains followed the Paleopathology Association Statement of Ethical Principles. The skeleton was cleaned and macroscopically investigated using a magnifying glass with 10x magnification. Apart from other pathological changes on the bones, the mandible showed distinct lytic lesions of the alveoli of the anterior dentition. This bone was brought to the German Archaeological Institute (DAI) for further diagnostic procedures. For detailed photographic documentation, the digital microscope Hirox KH-870,031 was used.

Plain radiography was undertaken by contact radiography with a Faxitron X-ray cabinet (Faxitron 43805 N by Hewlett-Packard at the DAI) followed by micro-CT scanning (Bruker™ SkyScan 2211 X-Ray Nanotomograph) with an image spatial resolution of 50 μm at the Max Planck Institute for Evolutionary Anthropology. The radiographs were evaluated for the lesions´ predominant appearance, either radiolucent, radiopaque, or mixed, and its transition to the surrounding bone. An undecalcified histological sample was taken from the alveolus of tooth 32 in a sagittal plane with a slice thickness of 60-µm. Using plain and polarizing microscopy (Leica Microscope DM R), it was assessed for resorptive processes and the nature of the macroscopically visible mineralized tissue within the lesion.

## Results

### Macroscopic and Digital Microscopic Examination

**Fig. 2:**
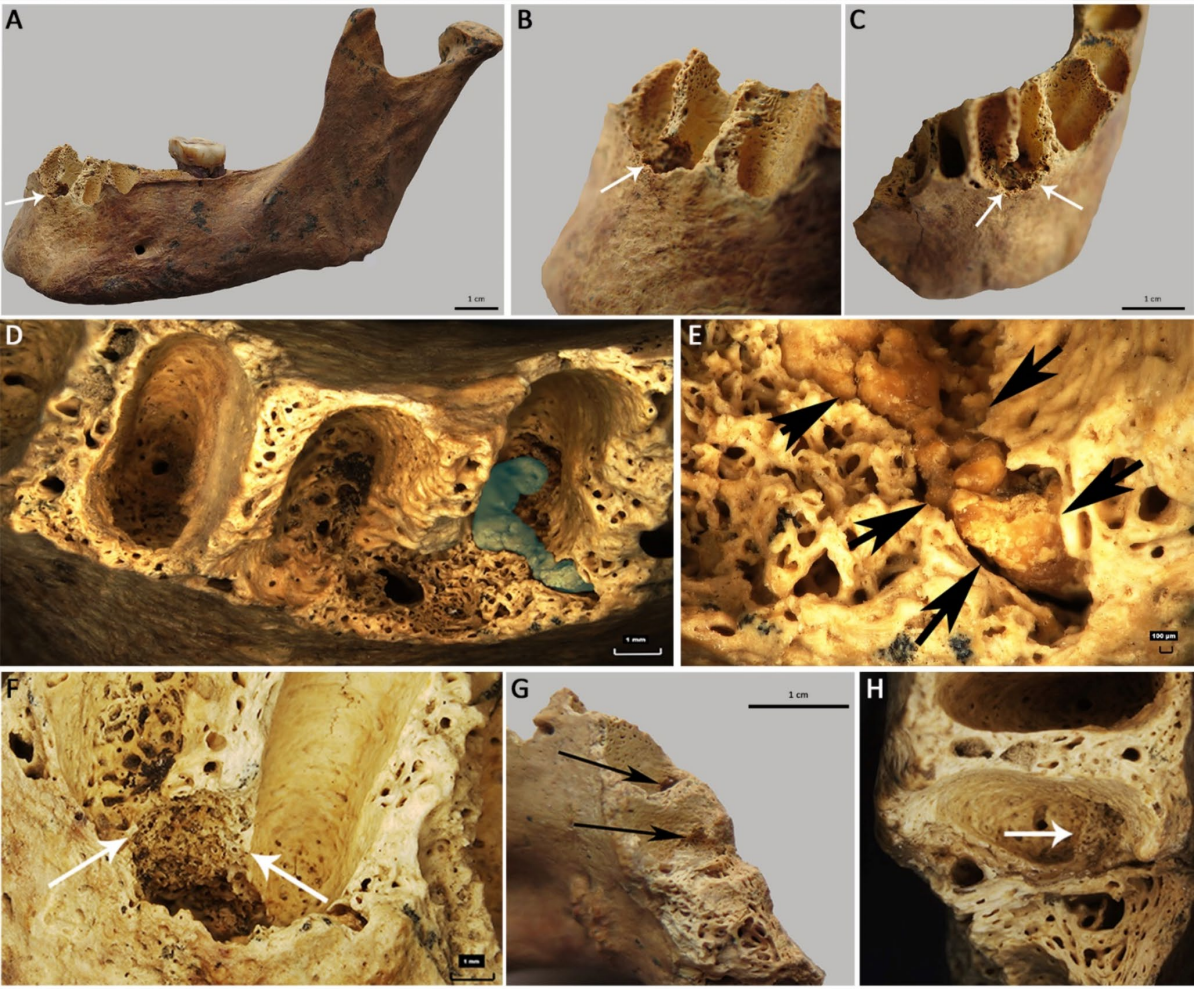
Macroscopic (**A**-**C**, **G**) and digital microscopic (**D**-**F**, **H**) images of the lesion in alveoli 32–42. Digital microscope, Hirox KH-870031. **A**) Left mandible with the lesion between the alveoli 32 and 31 (arrow). **B**) Close-up of the lesion (arrow). **C**) View of the lesion (arrows) from anterior-superior. **D**) Close-up of the lesion with dense particles (in blue) on the base of alveolus 32. **E**) Conglomerate of roundish particles (arrows) embedded in fine porous bone. **F**) Destruction of the alveolar septum and replacement by fine porous bone (arrows). **G**) Postmortem broken part of the mandible in the area of the alveolus 42. The upper arrow indicates the lesion in alveolus 41, and the lower arrow in alveolus 42. **H**) Superior view into alveolus 42 with a lesion on the anterior base of the alveolus (arrow). (Credits **A**-**C**, **G**: J. Gresky, **D**-**F**, **H**: M. Frotscher)

The base of the alveolus of tooth 32 shows a roundish 5 × 6 × 6 mm destructive defect which extends inferior of the alveolar septum into the base of alveolus 31 (Fig. 2A-F). The lesion is sharply demarcated, its surface is very fine porous, consisting of small irregular trabeculae (Fig. 2D-F) in contrast to the original alveolar surfaces. The distal two-thirds of the floor of alveolus 32 contain a conglomerate of roundish particles that have a slightly lighter color than the surrounding bone (Fig. 2D, E). They are embedded within the fine porous bone of the alveolus´ floor. Inferior to the alveolar septum between alveoli 31 and 32 to the lingual wall there is an approximately 2 × 1 mm hollow cavity (Fig. 2D, F). The labial wall in the septum region is thinner than the other labial walls and its compact bone is replaced by fine porous bone which is also visible on the external surface of the mandible in an area of 3 × 2 mm. The anterior external wall of the alveoli 31 and 32 is broken postmortem.

The mesial-labial wall of alveolus 41 shows an approximately 3 × 2 mm oval cavity exhibiting a fine porous bone structure on its base (Fig. 2G, upper arrow). A similar but 2 × 1 mm large cavity is visible on the mesial-labial wall of the alveolus of tooth 42 (Fig. 2G, lower arrow, H, arrow).

### Plain Radiology and Micro-Computed Tomography Examination

**Fig. 3:**
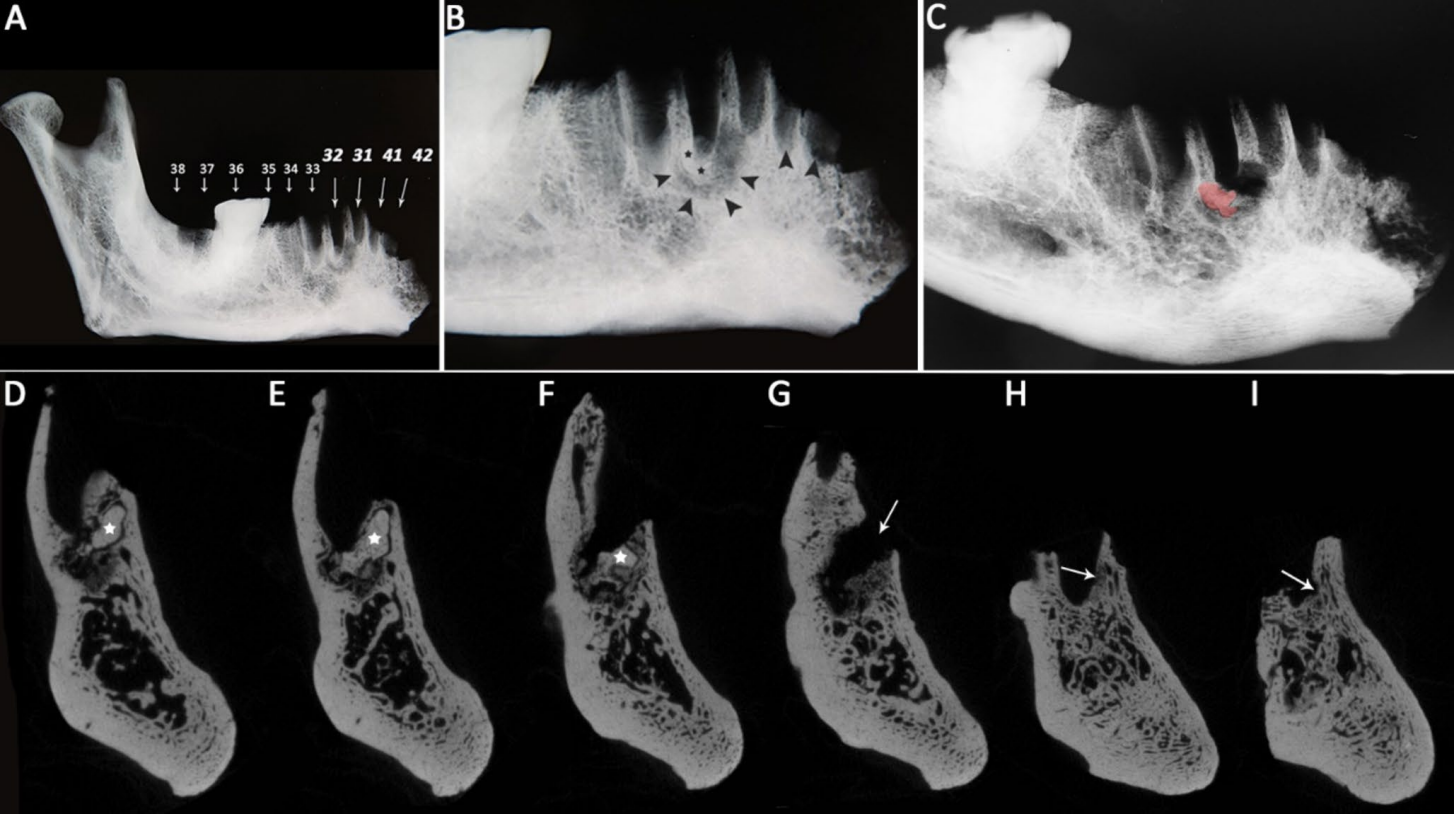
**A**-**C**) Plain radiographs and **D**-**I**) Micro-CT scans of the left mandible of the 30-40-year-old female of Budyonnovsk 10, burial mound 7, burial 2. **A**-**C**) Faxitron 43805 N by HewlettPackard. **A**, **B**) lateral beam path, 35 kV, 3.5 min, **C**) posterior-anterior beam path, 40 kV, 3.5 min. **A**) numbering of alveoli after FDI scheme, **B**) demarcated radiolucent areas periapical of alveoli of teeth 32 to 42 (arrows), nodular radiopaque deposit (stars). **C**) alveoli of the anterior teeth, the nodular radiopaque deposit marked in light red. **D**-**I**) SkyScan 2211, SkyScan 2211 X-Ray Nanotomograph by Bruker^TM^, 110 kV 170 µA, sagittal sections of the alveoli 32–42 of the mandible. Nodular radiopaque deposit (stars), radiolucent areas periapical of alveoli (arrows). **D**) septum between alveolus 33 and 32, the nodular radiopaque deposit is embedded into the bony wall, **E**) alveolus 32, **F**) lateral region of the septum between alveolus 32 and 31, **G**) mesial region of the septum between alveolus 32 and 31, **H**) alveolus 41, **I**) alveolus 42. (Credits: **A**-**C**: J. Gresky, **D**-**I**: A. Stoessel)

Plain radiography analyses show the localized lesions periapical of alveoli 32, 31, 41, and 42 (written in bold italics Fig. 3A). In lateral view (Fig. 3A, B) a radiolucent roundish area with a well-defined border surrounds the tip of alveolus 32 (Fig. 3A, B, arrows) and touches the distal border of alveolus 31. The entire lesion is radiolucent except for a nodular radiopaque deposit (Fig. 3B stars). This deposit is located in the distal half of the lesion, defining it as a mixed lesion. In the alveoli of teeth 41 and 42 only small lytic lesions which show a less well-defined border are visible (Fig. 3B, arrows). In the posterior-anterior beam path (Fig. 3B, C), the extent of the nodular deposit is better visible (Fig. 3C, colored in light red).

Micro-computed tomography images (Fig. 3D-I) show the radiopaque deposit which is solely located in the alveolus of tooth 32 (Fig. 3D-F). The lytic lesion of alveolus 32 continues into alveolus 31 visible as resorption of the compact bone of the lingual more than the labial sides. The lesion is partly filled by very fine porous bone which is less radiopaque than the original bone. The radiolucent lesions on the mesial walls of alveoli 41 and 42 (Fig. 3H, I, arrows) are very small, solely radiolucent, and not interconnected with each other or the large lesion.

### Light Microscopic Examination

**Fig. 4:**
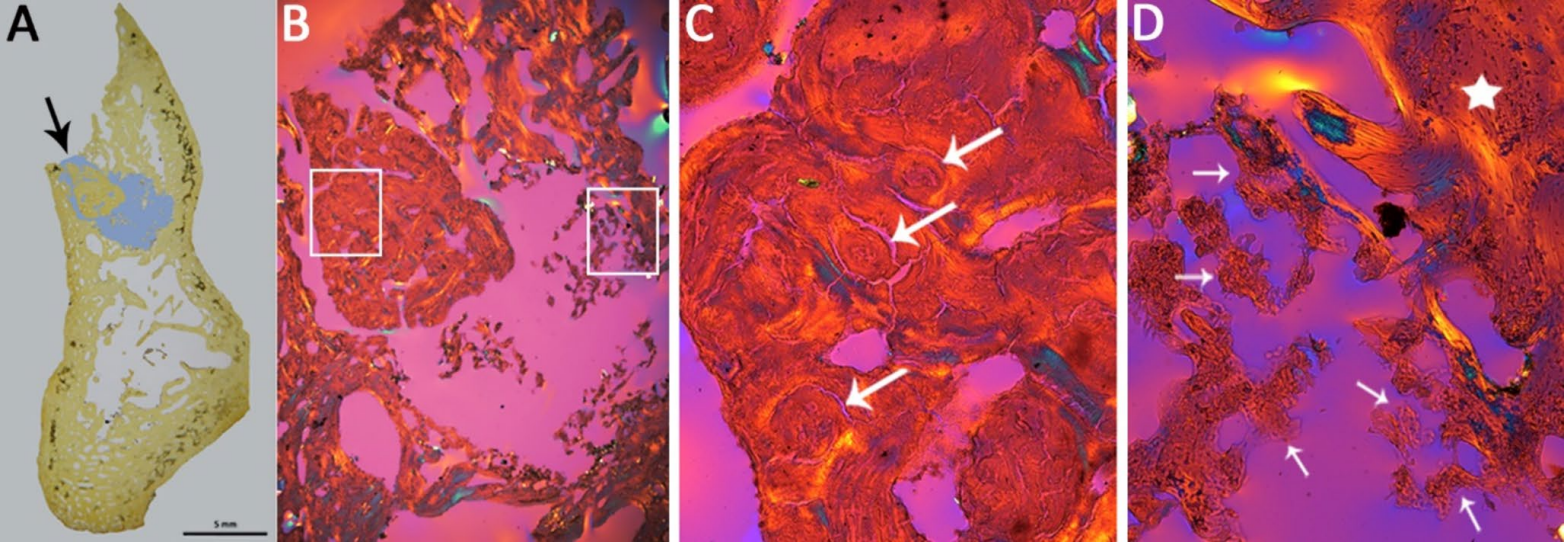
Microscopic images of the alveolus 32 of the mandible of the 30–40 years old female of Budyonnovsk 10, burial mound 7, burial 2. 60-µm thick section viewed in polarized light using quartz as a compensator. **A**) Sagittal section of the alveolus 32 in plain light. The resorptive lesion is colored blue, including small islands of woven bone and a conglomerate of a hypocellular matrix (arrow). **B**) 16× magnification of the lesion with the dense conglomerate of a hypocellular matrix (left) and the interspersed areas of woven bone (right). **C**) Conglomerate of a hypocellular matrix with heterogeneous structure, including roundish particles (arrows), 100× magnification. **D**) Bulky trabeculae of woven bone (arrows) attached to remnants of original trabecular bone (stars), 100× magnification. (Credits: J. Gresky)

A thin section was prepared on the level of the distal region of the alveolus of tooth 32 in a sagittal direction (Fig. 4). This section shows the resorption of the compact bone, the void area which probably was filled by fibrous tissue and which is interspersed by small islands of woven bone (Fig. 4A, blue filling). A large conglomerate of hypocellular matrix is located in the labial area of the lesion (Fig. 4A, arrow). It is distinctly demarcated from the surrounding bone. Its mass is not homogeneous but shows areas of defined roundish particles and irregular cement lines (Fig. 4C) suggesting single round particles being connected by additional hypocellular materials, by their shape resembling a ginger root. The borders of the resorptive lesion do not show clear evidence of osteoclastic activity. Instead, the woven bone seems to be attached to the walls of the original compact bone (Fig. 4D). The lack of Howship lacunae might be due to poor preservation.

## Discussion

### Limitations of the Study

There are two main limitations in diagnosing diseases in skeletal remains from archaeological sites: Taphonomic processes can alter bone and destroy formerly present pathological changes or they might simulate characteristics of diseases. In the presented case, postmortem destruction of the lesion´s walls is visible, however, this does not hinder the diagnosis based on other distinct features: Replacement of compact by woven bone and the presence of cementum-like materials are particularly clearly visible in the microscopic analysis. The second limitation is the missing soft tissue, laboratory values, and the patient´s clinical history. Without this information, diagnosing is challenging, but not impossible.

### Differential Diagnosis and Probable Diagnosis

Applying differential diagnosing, we were able to narrow down the diagnosis, even in a 4500-year-old skeleton: The combination of woven bone and cementum-like materials in microscopic evaluation could encompass odontogenic fibroma, cementoblastoma, ossifying fibroma, and cemento-osseous dysplasia [1].

The odontogenic fibroma is a very rare neoplasm, mainly located in the region of the mandibular premolars [18]. It comprises soft tissue components (odontogenic epithelium and fibrous stroma) rather than mineralized materials like woven bone or cementum [18]. If dysplastic cementum or osteoid is present, it is only focal within the lesion and does not show a conglomerate [18].

A cementoblastoma is unlikely as there is no evidence of blending with the root of a tooth or root resorption [19]; instead, the cementum-like tissue is separate from the roots of the teeth. Furthermore, the preferred location of the permanent first mandibular molar does not match the localization of the resorption in the presented case [19].

Ossifying fibroma (OF) cannot be excluded by its composition of mineralized materials of varying appearances [20]. However, its preferred location in the posterior mandible [21], its often-occurring jaw expansion, and larger size lesions [22] do not match the features of the presented case. While radiographically the presence of a well-defined border rather points to OF than cemento-osseous dysplasia (COD), the mixed radio-opacity is characteristic of COD [22]. Furthermore, OF cases are less often related to tooth apices than COD [22].

Cemento-osseous dysplasia is the most frequently occurring fibro-osseous lesion of the jaws [23] and is located in the periapical region of the tooth-bearing areas, predominantly of the mandible [24, 25]. Its main characteristic feature is the replacement of normal bone by metaplastic bone mixed with fibrous tissue [24] in close association with tooth apices. Radiology and computed tomography images of this case show a mixed lesion with a well-defined radiolucent rim around the radiopacity, characteristic of an intermediate stage [22]. The large lesion which shows a fusion of periapical lesions of alveoli 32 and 31 shows more mineralization than the exclusively radiolucent lesions of alveoli 41 and 42.

Apart from the missing cellular fibrous tissue caused by taphonomic reasons, microscopically, woven bone and cementum-like material are detectable. One important aspect in favor of the diagnosis of COD is the missing fusion of the hard tissue components with the roots of the teeth. The latest edition of the WHO classification [26] lists four different subtypes of COD: the best fitting is the periapical COD which has multiple foci in the region of the lower incisors.

### Contextualizing of the Oldest Case of COD from the Bronze Age

The presented case of COD in a 30-40-year-old female individual from the Bronze Age burial mound cemetery of Budyonnovsk in the Northern region of the Caucasus is the oldest evidence of such a disease in humans so far. Within this cemetery, buried in close vicinity, a 22-25-year-old male individual (burial 14) showed a similar pathology, a unifocal cemento-osseous dysplasia of the alveolus 43 [6]. Both individuals share similar grave goods and types of grave construction indicating that they might have been buried during the same period, the Late Catacomb, more precisely, the East Manych Catacomb Culture, which dates to 2500 − 2300 BC. Archaeologically, burial 2 is earlier than burial 14, but the chronological gap is small, and as such both individuals could have lived contemporarily or individual 14 was born later than individual 2. The probability that two individuals sharing the diagnosis of cemento-osseous dysplasia and who are buried in one burial mound might be biologically related, is worth being discussed. Although a familial basis of florid cemento-osseous dysplasia is known [27], familial cases of periapical are less often reported [28]. Genetic analysis is planned to detect a possible biological relationship between the two affected individuals and depending on the bone preservation to search for hotspot mutations involving the RAS-MAPK signaling pathway, which were identified to be responsible for 28% of COD cases [5].

The benefit of samples from archaeological skeletons is the availability of bone tissue which is neither decalcified nor scarce because it has not to be taken from a living person. Therefore, we hope to get enough tissue samples to perform a sufficient genetic analysis. Furthermore, unconventional views on the lesions are possible using samples of paleopathological research: They can provide a macroscopic view of the lesion and surface details, which is impossible in a living patient.

The oldest two cases of COD from the Bronze Age show that this disease has a long, yet unknown, history of occurrence and did not seem to have changed in its appearance over the last 4500 years.

## Data Availability

No datasets were generated or analysed during the current study.

## Declarations

### Code Availability

This declaration is not applicable

## References

[1] Barnes L (2007) Weltgesundheitsorganisation, and International Agency for Research on Cancer. Pathology and Genetics of Head and Neck Tumours. World Health Organization Classification of Tumours 9, IARC, Lyon

[2] Gresky J (2020) Fibro-osseous processes (FOPs) of the Craniofacial Skeleton: a neglected. Entity Paleopathology? HOMO 71(4):281–297 33146662 10.1127/homo/2020/1277

[3] Gresky J (2023) Fibro-osseous processes of the craniofacial skeleton: what human skeletal remains from archaeological contexts can contribute to clinical research. Osteol 32(03):S9–S10

[4] Baumhoer D, Haefliger S, Ameline B, Hartmann W, Amary F, Cleven A, Klein MJ, Thompson LDR, Harder D, O’Donnell P (2022) Ossifying fibroma of non-odontogenic origin: a fibro-osseous lesion in the craniofacial skeleton to be (re-)considered. Head Neck Pathol 16:257–267 34173971 10.1007/s12105-021-01351-3 PMC9018933

[5] Haefliger S, Turek D, Andrei V, Alborelli I, Calgua B, Ameline B, Harder D, Baumhoer D (2023) Cemento-osseous dysplasia is caused by RAS-MAPK activation. Pathol 55(3):324–328 10.1016/j.pathol.2022.10.006 36707318

[6] Gresky J, Kalmykov A, Berezina N (2018) Benign fibro-osseous lesion of the mandible in a middle bronze age skeleton from Southern Russia. Int J Paleopathol 20:90–97 29496222 10.1016/j.ijpp.2017.09.001

[7] Ruffer MA (1921) Studies in the palaeopathology of Egypt. University of Chicago Press, Chicago

[8] Moodie RL (1923) Paleopathology. An introduction to the study of ancient evidences of disease. University of Chicago Press, Chicago

[9] editor Grauer AL (2012) A companion to Paleopathology. Blackwell Companions to Anthropology 14. Wiley-Blackwell, Chichester, West Sussex, Malden, MA

[10] Bos KI, Harkins KM, Herbig A, Coscolla M, Weber N, Comas I, Forrest SA, Bryant JM, Harris SR, Schuenemann VJ et al (2014) Pre-columbian mycobacterial genomes reveal seals as a source of New World human tuberculosis. Nature 514:494–497 25141181 10.1038/nature13591 PMC4550673

[11] Majander K, Pla-Díaz M, Du Plessis L, Arora N, Filippini J, Pezo-Lanfranco L, Eggers S, González-Candelas F, Schuenemann VJ (2024) Redefining the Treponemal History through pre-columbian genomes from Brazil. Nature 627(8002):182–188 38267579 10.1038/s41586-023-06965-x PMC10917687

[12] Gresky J, Frotscher M, Dorn J, Scheelen-Nováček K, Ahlbrecht Y, Jakob T, Schönbuchner T, Canalejo J, Ducke B, Petiti E (2024) The Digital Atlas of Ancient Rare diseases (DAARD) and its relevance for current research. Orphanet J Rare Dis 19:277 39044201 10.1186/s13023-024-03280-0 PMC11267669

[13] Rohrlach AB, Rivollat M, de-Miguel-Ibáñez P, Moilanen U, Liira A-M, Teixeira JC, Roca-Rada X, Armendáriz-Martija J, Boyadzhiev K, Boyadzhiev Y et al (2024) Cases of trisomy 21 and trisomy 18 among historic and prehistoric individuals discovered from ancient DNA. Nat Commun 15:1294 38378781 10.1038/s41467-024-45438-1 PMC10879165

[14] Ferembach D, Schwidetzky I, Stloukal M (1979) Empfehlungen für die Alters- Und Geschlechtsdiagnose am Skelett. HOMO 30:1–32

[15] Buikstra JE, Ubelaker DH (eds) (1994) Standards for data collection from human skeletal remains. Arkansas Archeological Survey Research Series 44, Fayetteville, AR

[16] Brothwell DR (1981) Digging up bones: the excavation, treatment, and study of human skeletal remains. Cornell University Press, Ithaca

[17] Brooks S, Suchey JM (1990) Skeletal age determination based on the os pubis: a comparison of the Acsádi-Nemeskéri and Suchey-Brooks methods. Hum Evol 5(3):227–238

[18] Philipsen HP, Reichart PA, Sciubba JJ, van der Waal I (2007) Odontogenic fibroma. In: Barnes L (ed) Weltgesundheitsorganisation, and International Agency for Research on Cancer. Pathology and Genetics of Head and Neck tumours. World Health Organization Classification of Tumours 9. IARC, Lyon, p 315

[19] van der Waal I (2007) Cementoblastoma. In: Barnes L (ed) Weltgesundheitsorganisation, and International Agency for Research on Cancer. Pathology and Genetics of Head and Neck tumours. World Health Organization Classification of Tumours 9. IARC, Lyon, p 318

[20] Slootweg PJ, El-Mofty SK (2007) Ossifying fibroma. In: Barnes L (ed) Weltgesundheitsorganisation, and International Agency for Research on Cancer. Pathology and Genetics of Head and Neck tumours. World Health Organization Classification of Tumours 9. IARC, Lyon, pp 319–320

[21] Brannon RB, Fowler CB (2001) Benign fibro-osseous lesions: a review of current concepts. Adv Anat Pathol 8:126–143 11345237 10.1097/00125480-200105000-00002

[22] Su L, Weathers DR, Waldron CA (1997) Distinguishing features of focal cemento-osseous dysplasia and cementoossifying fibromas. II. A clinical and radiologic spectrum of 316 cases. Oral Surg Oral Med Oral Pathol Oral Radiol Endod 84:540549 10.1016/s1079-2104(97)90271-7 9394387

[23] Nelson BL, Phillips BJ (2019) Benign fibro-osseous lesions of the head and neck. Head Neck Pathol 13:466–475 30887390 10.1007/s12105-018-0992-5 PMC6684826

[24] Slootweg PJ (2007) Osseous dysplasias. In: Barnes L (ed) Weltgesundheitsorganisation, and International Agency for Research on Cancer, editors. Pathology and Genetics of Head and Neck tumours. World Health Organization Classification of Tumours 9. IARC, Lyon, p 323

[25] El-Mofty SK, Nelson B, Toyosawa S, Wright JM (2017) Cementoosseous dysplasia. In: El-Naggar AK, Chan JKC, Grandis JR, Takata T, Slootweg PJ (eds) WHO classification of Head and Neck Tumours, 4th edn. IARC, Lyon, pp 254–255

[26] Haefliger S, Baumhoer D (2023) The new WHO classification of jaw tumours. Pathol 44(4):240–249 10.1007/s00292-023-01195-4 PMC10322770 37179260

[27] Nel C, Yakoob Z, Schouwstra C-M, vanHeerden WF (2021) Familial florid cemento-osseous dysplasia: a report of three cases and review of the literature. Dento Maxillo Facial Radiol 50:20190486 10.1259/dmfr.20190486 PMC7780834 32315206

[28] Thakkar NS, Homer K, Sloan P (1993) Familial occurrence of periapical cemental dysplasia. Virchows Archiv Pathol Anat 423:233–236 10.1007/BF01614776 8236819

